# Predictors of Support-Seeking During Stress Discussions of Older Adult Couples

**DOI:** 10.3390/bs15101393

**Published:** 2025-10-15

**Authors:** Yuxi Xie, Brooke C. Feeney

**Affiliations:** 1 Department of Psychology, University of Macau, Taipa, Macau, China; yuxixie@um.edu.mo; 2 Department of Psychology, Carnegie Mellon University, Pittsburgh, PA 15213, USA

**Keywords:** support-seeking, instrumental support, emotional support, attachment orientation, older adulthood

## Abstract

Despite its central role in fostering effective social support, support-seeking behavior has received limited empirical attention—particularly among older adults, who have heightened needs for support due to age-related cognitive and physical decline. This study identified and examined key predictors of four types of support-seeking behaviors—direct and indirect instrumental and emotional support-seeking. Long-term married couples, with at least one partner aged 65 or older, participated in a laboratory-based discussion about a personal life stressor, during which support-seeking behaviors were coded. We examined both the support-seeker’s and support-provider’s attachment orientation, as well as the support-seeker’s stress level and relationship quality (e.g., satisfaction, commitment, and trust), as predictors of observed support-seeking behaviors. Results indicated that greater stress and higher relationship quality were associated with more direct instrumental support-seeking, while lower relationship quality and greater attachment insecurity in both partners predicted more indirect instrumental and emotional support-seeking. However, support-seekers also showed more direct emotional support-seeking with avoidantly attached partners, possibly as a compensatory effort to elicit needed support. This study contributes to the limited literature focusing on support-seeking behaviors, particularly in older adulthood, and has implications for interventions aimed at promoting effective communication and support-seeking.

## 1. Introduction

In everyday life, people encounter situations in which they may benefit from turning to others for support (Feeney, 2004). For example, people sometimes need a specific form of task assistance (i.e., instrumental support), or emotional comfort and reassurance (i.e., emotional support)—and they are most likely to rely on those closest to them to provide this support (e.g., romantic partners). However, this support process can go awry when potential support partners do not understand that the person needs help, the specific type of support wanted or needed, or how to provide the needed support (Bolger et al., 2000). This points to the need for a potential recipient of support to engage in effective support-seeking.

Despite its importance, research on support-seeking in close relationships has been scarce relative to research on support provision. Most research on social support treats support-seekers as passive recipients of support, ignoring support-seeking altogether. However, support-seekers can cultivate effective support in a variety of ways (Feeney & Collins, 2015; Forest et al., 2021; Kuang & Wang, 2023; Schwaninger et al., 2021). For example, support-seekers can facilitate the social support process by openly expressing their concerns and the kind of help they need (e.g., instrumental or emotional), and by being open and receptive to support. Indeed, past research shows that direct support-seeking (i.e., explicitly asking for help or expressing one’s needs to a partner) elicits more helpful forms of support (Barbee & Cunningham, 1995; Don & Hammond, 2017), while indirect support-seeking (i.e., attempting to elicit support without openly stating one’s needs) has been linked to negative responses from the support-provider and decreases in effective support provision (Barbee & Cunningham, 1995; Don et al., 2013, 2019).

However, not all individuals are equally likely to seek support from their romantic partners, and even among those who do, the strategies (direct vs. indirect, instrumental vs. emotional) they use can vary widely. While a substantial body of research has examined predictors of support provision in close relationships (e.g., Berli et al., 2021; Iida et al., 2008), relatively few studies have investigated the factors that predict support-seeking behaviors (Feeney & Collins, 2015). Understanding who seeks support and how they do so is critical for advancing research on social support (given the often-overlooked role of support-seeking) and for informing interventions aimed at improving communication and coping in intimate relationships. Moreover, the limited existing research on support-seeking has rarely focused on older adult couples—a population that warrants greater attention given that social support becomes increasingly important in later adulthood as individuals face age-related physical and cognitive declines (Antonucci et al., 2014; James et al., 2011).

To address these gaps in theory and past research, this study identified and examined key predictors of support-seeking behaviors—specifically, direct instrumental support-seeking, direct emotional support-seeking, indirect instrumental support-seeking, and indirect emotional support-seeking—that occur when couple members are discussing personal life stressors and could potentially benefit from receiving support. We examined both the support-seeker’s and support-provider’s attachment orientation, as well as the support-seeker’s stress level and relationship quality (e.g., satisfaction, commitment, and trust), as predictors of observed support-seeking behaviors. We focused this investigation on older adult couples, an age group often overlooked in the social support literature. Each predictor is described in detail below.

### 1.1. Support-Seeker’s Stress Level

Previous research has shown that support-seekers’ stress level is associated with their support-seeking behaviors. For example, people report a greater desire to be with their partner during stressful situations (Azzi et al., 2022), and affiliation in times of stress has been strongly documented (Taylor, 2010). Consistent with previous research in young adult samples showing that support-seekers engaged in more direct support-seeking behaviors when they rated their problem as more stressful (Collins & Feeney, 2000; Li & Yang, 2009; Simpson et al., 1992), we expected that older adults will also exhibit more direct and fewer indirect support-seeking behaviors when experiencing greater stress.

### 1.2. Support-Seeker’s Attachment Orientation

Prior research also has considered attachment orientation as a predictor of support-seeking (e.g., Loeb et al., 2021; Ognibene & Collins, 1998; Snyder et al., 2024). Both self-report (Florian et al., 1995; Francois-Walcott et al., 2024; Mikulincer & Florian, 1995) and observational (Simpson et al., 1992) studies with younger participant samples show that more securely attached individuals seek more support when distressed, while more avoidantly attached individuals seek less support when distressed. These findings support attachment theory’s postulates that distress experienced either directly or vicariously can activate mental models of self (beliefs about the worthiness of the self to receive care and support from others) and others (beliefs about others’ dependability and reliability). These mental models underlie secure, anxious, and avoidant attachment orientations (Bowlby, 1973) and lead people to behave in ways consistent with their mental representations of self and others. Interestingly, existing research indicates that support-seekers high in attachment anxiety tend to ambivalently seek both proximity to and distance from their partners in experimentally induced stressful situations (Dewitte et al., 2008). This ambivalent behavioral pattern—behaviors that are clingy and demanding while also showing angry resistance (George & Solomon, 1996; Simpson et al., 2002)—theoretically stems from a history of inconsistent responsiveness from attachment figures (Collins & Feeney, 2000; Simpson et al., 2002).

Consistent with this previous research with younger samples, we expected more securely attached older adults to directly seek support from their partners. We also expected more avoidantly attached participants to engage in less direct support-seeking of any form (emotional or instrumental) and to seek support indirectly if at all, given their discomfort with vulnerability and mental representations of others as unreliable. We expected more anxiously attached older adults to behave ambivalently, without a consistent support-seeking pattern, given their history of experiences with inconsistent caregivers. We further expected that older adults would be most likely to act in ways consistent with their attachment-based mental representations in increasingly stressful situations, given attachment theory’s postulate that mental representations most strongly drive behaviors when the attachment system is strongly activated (Bowlby, 1988).

### 1.3. Support-Provider’s Attachment Orientation

The support-provider’s attachment orientation also should strongly predict support-seeking behavior. Surprisingly, this has not been fully investigated in prior research, except for one study showing that support-seekers tended to distance themselves from partners who reported high levels of anxiety in stressful contexts (Campbell et al., 2001). However, prior research has shown that attachment orientation strongly predicts support-giving behavior, as well as characteristics that underlie effective support provision, such as skills, personal resources, and motivation (Feeney & Collins, 2001, 2015). Because the support-provider’s attachment orientation is critical to the success of support interactions, it should also be a key predictor of a partner’s support-seeking behaviors enacted toward the support-provider.

Given prior research linking secure attachment orientation to more sensitive and responsive support provision (Collins & Feeney, 2000; Feeney & Collins, 2001; Simpson et al., 1992), we predicted more direct emotional and instrumental support-seeking toward securely attached support-providers. Moreover, given that individuals with either an avoidant or anxious attachment orientation are less sensitive and responsive support-providers (e.g., Feeney & Collins, 2001; Simpson et al., 1992), we predicted less direct and more indirect support-seeking toward these partners, particularly toward avoidant partners who dislike expressions of vulnerability. We also expected greater experiences of stress (greater attachment system activation) to exacerbate these tendencies. Furthermore, we expected significant interactions between a support-seeker’s and support-provider’s attachment orientations: we expected less direct and more indirect support-seeking of any form (instrumental or emotional) when both partners are insecure.

### 1.4. Support-Seeker’s Perceived Relationship Quality

Relationship context should also be an important predictor of support-seeking toward a specific partner. Seeking support is often not easy as there can be associated costs. For example, it is common for people to feel uncomfortable even asking for help from intimate others (Hobfoll & Lerman, 1988) because support-seeking can make individuals feel weak, indebted, or like a burden (Clark, 1983). Also, help may be threatening if it implies an inferior–superior relationship between support-seeker and provider, triggering a conflict between self-reliance (autonomy) and dependence on others, which may harm the support-seeker’s self-esteem (Merton & Kitt, 1950). High-quality relationships characterized by commitment and trust could reduce or eliminate concerns about relying on an intimate partner for support. For instance, because trust involves confidence in a partner’s responsiveness to one’s needs as well as a willingness to put oneself at risk (Holmes, 1991), relationship trust may facilitate support-seeking. Other features of relationship quality (e.g., commitment, satisfaction, and intimacy) should similarly facilitate support-seeking because of the strong bond it signals between partners. Because support is expected to be forthcoming in high-quality relationships, we hypothesized that higher perceived relationship quality should predict more direct seeking of both emotional and instrumental support.

### 1.5. Study Hypotheses

In summary, we examined both the support-seeker’s and support-provider’s attachment orientation, as well as the support-seeker’s stress level and relationship quality (e.g., satisfaction, commitment, and trust), as predictors of observed support-seeking behaviors (objectively coded from video-recorded interactions). We hypothesized that older adults would engage in more direct and less indirect support-seeking behaviors when experiencing higher stress or having higher relationship quality. Securely attached individuals and those with securely attached partners were expected to seek more direct and less indirect support. In contrast, individuals with greater attachment insecurity, as well as those with insecurely attached spouses, were expected to seek less direct and more indirect support, particularly under stress. Furthermore, we anticipated significant interactions between the support-seeker’s and support-provider’s attachment orientation such that less direct and more indirect support-seeking of any form (instrumental or emotional) would occur when both partners were insecure.

## 2. Materials and Methods

### 2.1. Participants

Participants were 271 older adult married couples with at least one member over age 65 and recruited as part of a larger study of relationships in late adulthood conducted in Pittsburgh, PA. Couples were recruited through a random digit dialing procedure that targeted households with a resident over age 65. If both couple members were over age 65, one member was randomly designated to be the “target participant” whose support-seeking would be assessed, and the other member implicitly took the role of a potential support-provider (who we refer to as the “spouse”). If only one couple member was 65 or older, that person was designated as the “target participant” as the larger investigation focused on older adulthood. Of the 271 couples enrolled, 211 completed the observational session in which support-seeking behaviors were assessed. Mean age of participants was 70.1 (SD = 6.8), with target participants (support-seekers) slightly older (M = 70.9, SD = 5.6) than spouses (support-providers; M = 69.4, SD = 7.7). Couples had been married for 41 years on average (SD = 14.1). Demographics included 83% White American, 11% Black or African American, 0.5% Hispanic or Latino American, 0.5% Asian American, and 4% other race or preferred not to say. There were no exclusionary criteria for selecting current study participants from the original sample. Participants provided informed consent prior to participation, and the study was approved by Carnegie Mellon University’s institutional review board (Number: HS14-100). Participants were financially compensated for their participation. Sample size was determined a priori based on power analysis for the larger study. For this investigation, we used G*Power 3.1 to conduct a post hoc analysis of achieved power for a linear multiple regression (fixed model, R^2^ increase) with six predictors, and an α error probability of 0.05. This study had 66% power to detect a small effect (f^2^ = 0.05) and 99% power to detect a medium effect (f^2^ = 0.15).

### 2.2. Procedure

Couples came to the research laboratory, one couple at a time, to complete baseline questionnaires, including assessments of attachment orientation and relationship quality.

Approximately one week later, each couple returned to the laboratory for an observational session in which several interactions were recorded unobtrusively as part of the parent project. This investigation is focused on a “stress discussion” between couple members, which is an ideal context to examine support-seeking behavior. Prior to the discussion, the target participant (in the role of a potential support-seeker) completed a pre-discussion questionnaire. In the questionnaire, they provided information about their most important worry, problem, or concern right now (that was not caused by the spouse), and rated how upsetting or stressful it was for them on a 5-point scale ranging from 1 = not at all to 5 = extremely. Then, the target participant was asked to discuss this stressor with the spouse. The discussion was recorded for seven minutes. Participants discussed stressors such as financial strains, a family member’s health, and job-related concerns.

### 2.3. Measures

#### 2.3.1. Baseline Assessments

Attachment orientation. The attachment orientation of both couple members was assessed using 24 items adapted from the Experiences in Close Relationships Scale (Brennan et al., 1998). Participants rated the extent to which they agreed with statements about their important relationships on a 7-point scale (1 = disagree strongly, 7 = agree strongly). This measure has two subscales: anxiety (α = 0.87; e.g., “I worry about being abandoned”) and avoidance (α = 0.85; e.g., “I prefer not to show people how I feel deep down”).

Relationship quality. Three components of the target participant’s (support-seeker’s) baseline relationship quality were assessed. Relationship satisfaction (α = 0.95) was assessed using four items from Van Lange et al. (1997) and two items from Collins and Read (1990) (e.g., “All things considered, how satisfied are you with your relationship?”) on 9-point rating scales with appropriate anchors (e.g., 0 = not at all, 8 = completely). Trust of spouse (α = 0.82) was assessed with eight items from Rempel et al.’s (1985) Trust Scale (e.g., “I feel that I can trust my spouse completely”; 1 = strongly disagree, 7 = strongly agree). Relationship commitment (α = 0.68) was assessed with six items from Rusbult et al. (1998) (e.g., “Do you feel committed to maintaining your relationship with your spouse?”; 0 = not at all, 8 = completely). These scales were chosen to capture distinct, theoretically meaningful dimensions of relationship quality; they are validated and widely used assessments in the literature on close relationships. A composite index of perceived relationship quality (α = 0.92) was calculated by standardizing and averaging the scores for each scale.

#### 2.3.2. Observed Support-Seeking Behaviors

Target participant’s support-seeking behavior was coded from the video recordings of each couple’s stress discussion. This observational method was chosen for its ability to capture real-time, objective support-seeking behaviors, reducing social desirability biases and recall errors that could occur with self-report assessments. Additionally, the use of predetermined coding criteria minimizes variability in interpretations of behaviors (Park & Lee, 2018). All discussions were coded by at least two independent observers who were blind to additional information about the participants. The observers were trained in using a detailed coding manual to standardize their assessments, and they were trained to reliability before coding. Four support-seeking behaviors were coded as follows: (1) direct emotional support-seeking (directly asking for support aimed at helping one to feel better about the stressor, ICC = 0.68); (2) direct instrumental support-seeking (directly asking for tangible or informational help/assistance in dealing with the concern, ICC = 0.79); (3) indirect emotional support-seeking (beating around the bush and appearing to want reassurance from the spouse but not asking for it directly, ICC = 0.70); and (4) indirect instrumental support-seeking (beating around the bush and appearing to want tangible assistance from the spouse but not asking for it directly, ICC = 0.56). According to general guidelines (Cicchetti, 1994), ICC values below 0.40 indicate poor reliability, 0.40–0.59 is fair, 0.60–0.74 is good, and 0.75–1.00 is excellent. Our codes show fair to excellent reliability. All behaviors were coded using a 5-point scale reflecting the frequency and quality or intensity of each form of support-seeking (1 = no occurrence, 5 = consistent and highest quality). For each support-seeking behavior, the average of two independent observations were used in data analysis.

### 2.4. Data Analyses

Multiple linear regression analyses were conducted in R (Version 2023.03.1+446) to predict the four types of support-seeking from the target participant’s (support-seeker’s) stress level (regarding the stressor discussed), attachment orientation, and perceived relationship quality, and from the spouse’s (support-provider’s) attachment orientation. All predictors were entered simultaneously. Variance inflation factors (VIFs) were all close to one, indicating no multicollinearity issues. We also added and tested interactions between the target participant’s attachment orientation and stress level, target participant’s attachment anxiety and avoidance, spouse’s attachment orientation and target participant’s stress level, spouse’s attachment anxiety and avoidance, and target participant’s and spouse’s attachment orientation using hierarchical regression.

## 3. Results

Descriptive statistics and correlations among all study variables are shown in Table 1.

The regression results for each support-seeking variable are summarized in Table 2.

### 3.1. Direct Instrumental Support-Seeking

As shown in Table 2, target participant’s (support-seeker’s) higher pre-discussion stress level and higher baseline relationship quality predicted greater direct instrumental support-seeking. There were no main effects of attachment orientation predicting direct instrumental support-seeking, and no significant interactions among those tested.

### 3.2. Direct Emotional Support-Seeking

**Figure 1:**
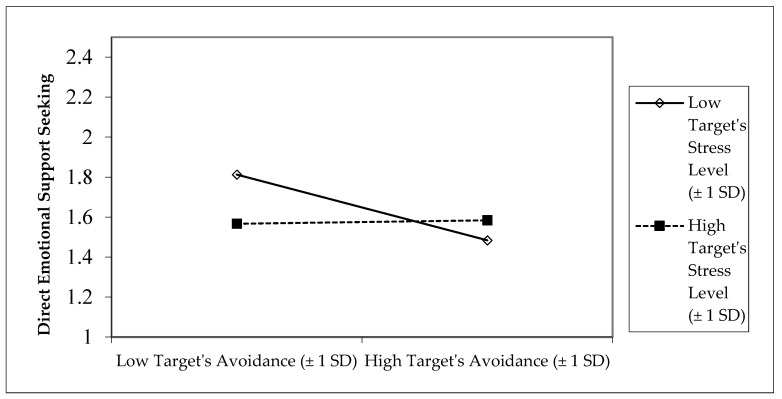
Interaction between target participant’s attachment avoidance and stress level predicting target participant’s direct emotional support-seeking.

Target participant’s (support-seeker’s) greater attachment avoidance predicted less direct emotional support-seeking. However, greater attachment avoidance of the spousal support-provider predicted more direct emotional support-seeking. Moreover, the interaction between target participant’s (support-seeker’s) attachment avoidance and stress level predicted their direct emotional support-seeking. As shown in Figure 1, target participants with low stress and low avoidance exhibited the greatest direct emotional support-seeking. Under low stress, target participants with high attachment avoidance displayed less direct emotional support-seeking. Under high stress, target participants maintained the same low level of direct emotional support-seeking regardless of their level of attachment avoidance (Figure 1). There were no other significant main effects or interactions predicting direct emotional support-seeking.

### 3.3. Indirect Instrumental Support-Seeking

**Figure 2:**
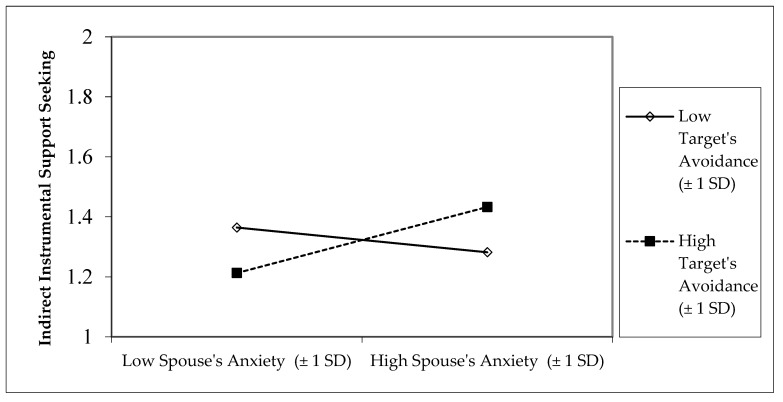
Interaction between target participant’s attachment avoidance and spouse’s attachment anxiety predicting target participant’s indirect instrumental support-seeking.

There were two significant interactions between target participant’s (support-seeker’s) and spouse’s (support-provider’s) attachment orientation predicting indirect instrumental support-seeking. First, an interaction between target participant’s (support-seeker’s) attachment avoidance and spouse’s (support-provider’s) attachment anxiety revealed that target participants with high attachment avoidance displayed more indirect instrumental support-seeking if their spouses had high attachment anxiety and less indirect instrumental support-seeking if their spouses had low attachment anxiety (Figure 2). There was also the reverse pattern for target participants with low attachment avoidance to exhibit less indirect instrumental support-seeking toward spouses who are high in attachment anxiety, and more indirect seeking toward spouses low in attachment anxiety.

**Figure 3:**
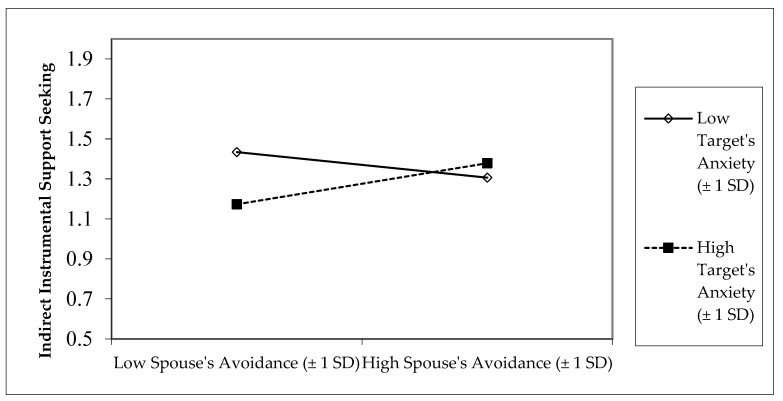
Interaction between target participant’s attachment anxiety and spouse’s attachment avoidance predicting target participant’s indirect instrumental support-seeking.

Second, an interaction between target participant’s (support-seeker’s) attachment anxiety and spouse’s (support-provider’s) attachment avoidance indicated that target participants (support-seekers) with high attachment anxiety displayed more indirect instrumental support-seeking if their spouses had high attachment avoidance, and less indirect support-seeking toward spouses who had low attachment avoidance (Figure 3). Figure 3 shows that the least indirect instrumental support-seeking occurred when spousal support-providers were low in attachment avoidance and support-seekers were high in attachment anxiety. There were no other significant main effects or interactions predicting indirect instrumental support-seeking.

### 3.4. Indirect Emotional Support-Seeking

**Figure 4:**
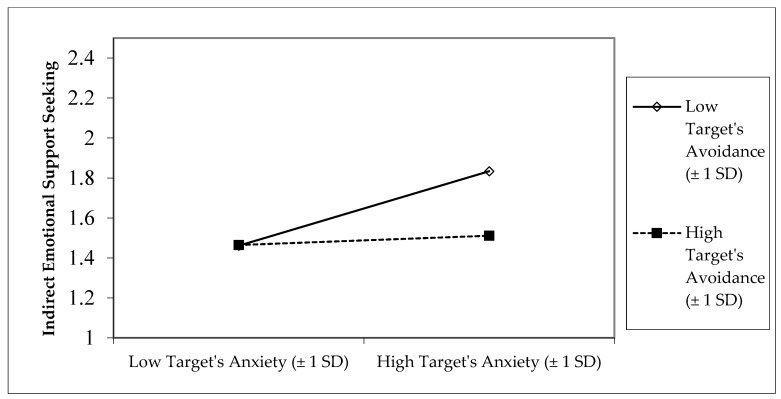
Interaction between target participant’s attachment anxiety and avoidance predicting target participant’s indirect emotional support-seeking.

Target participant’s (support-seeker’s) reports of greater relationship quality predicted less indirect emotional support-seeking. Also, there was a significant interaction between target participant’s (support-seeker’s) attachment anxiety and avoidance predicting their indirect emotional support-seeking. As shown in Figure 4, target participants with high attachment anxiety and low attachment avoidance (preoccupied attachment) displayed the most indirect emotional support-seeking. There were no other significant main effects or interactions predicting indirect emotional support-seeking.^1^

## 4. Discussion

This study contributes to the sparse literature on the predictors of support-seeking behaviors in older adult couples. We observed support-seeking behaviors during spousal discussions of stressors and tested hypotheses regarding several key predictors of support-seeking (i.e., both the support-seeker’s and support-provider’s attachment orientation, and the support-seeker’s stress level and relationship quality). We focused on four dimensions of support-seeking, including direct versus indirect, and both emotional and instrumental support-seeking.

### 4.1. Who Seeks Support Directly?

We expected that older adults who had higher stress levels and higher relationship quality would seek support more directly. As expected, older adults with higher stress levels and higher relationship quality showed greater direct instrumental support-seeking. These results are consistent with previous research showing that people have stronger desires to be with spouses in stressful situations (Azzi et al., 2022) and with the idea that higher relationship quality reduces concerns about weakness, vulnerability, indebtedness, and being a burden that can accompany support-seeking (Holmes, 1991).

We also expected that older adults would more directly seek support from securely attached spouses. Interestingly, inconsistent with predictions, older adults exhibited greater direct emotional support-seeking when their spouse (support-provider) had higher attachment avoidance. One explanation is that spouses with an avoidant attachment orientation have difficulties understanding support-seekers’ feelings; exhibit discomfort and disinterest in helping; and avoid providing intimate, emotional forms of support spontaneously and proactively (Shaver & Mikulincer, 2002). Thus, support-seekers may need to compensate by directly seeking emotional support from avoidant partners to get the support they need, illustrating a form of dyadic adaptation where support-seeking strategies are adjusted based on partner characteristics (Collins & Feeney, 2000).

Moreover, we predicted that older adults with higher attachment insecurity (particularly attachment avoidance) would not seek support directly. As predicted, older adults with higher attachment avoidance were less likely to directly seek emotional support. This is consistent with attachment theory and prior research (e.g., Simpson et al., 1992) linking attachment avoidance to avoidance of direct emotional support-seeking because of discomfort with sharing feelings, concerns, and vulnerabilities with partners. It is noteworthy, however, that we did not find this avoidant effect to exacerbate under high stress as we expected. It may be that attachment avoidance, independent of stress level, has an inhibiting effect on direct emotional support-seeking. Future research could observe support-seeking behaviors under varying stress levels to determine how stress interacts to impact support-seeking.

Furthermore, we did not find significant effects of either support-seeker’s or support-provider’s attachment orientation on direct instrumental support-seeking. One possible explanation is that direct emotional support-seeking is more vulnerable and emotionally charged than instrumental support-seeking, making it more relevant to attachment dynamics. This finding warrants replications in future research.

In sum, for predictors of older adults’ direct support-seeking, we found that higher stress and relationship quality predicted more direct instrumental support-seeking. Direct emotional support-seeking showed a more complex pattern: more direct emotional support-seeking occurred with avoidantly attached spouses, likely to compensate for their lack of proactive support; however, seekers’ own attachment avoidance inhibited their direct emotional support-seeking, underscoring how support-seeking behaviors are inherently dyadic, shaped by both the support-seeker’s and support-provider’s attachment orientation.

### 4.2. Who Seeks Support Indirectly?

We expected that older adults with greater attachment insecurity and those with more insecurely attached spouses would seek support more indirectly because of concerns about getting the support they need or desire. Consistent with predictions, support-seekers with higher attachment anxiety and lower attachment avoidance (preoccupied attachment) engaged in the most indirect emotional support-seeking. Anxious–preoccupied attachment is characterized by a strong desire to be close to relationship partners and to receive intensive emotional support from them (Bowlby, 1973). These individuals are highly motivated to obtain emotional support, warmth, and care, but their fear of rejection or neglect may lead them to seek support in a less direct manner. Engaging in more indirect emotional support-seeking may reflect an adaptive strategy, balancing their desire for support with the need to avoid overwhelming or alienating the partner. It remains for future research to replicate and test explanations for this finding.

Moreover, as predicted, more avoidant support-seekers sought more indirect instrumental support from spouses with high attachment anxiety. Similarly, more anxiously attached support-seekers sought more indirect instrumental support from spouses with high attachment avoidance. Consistent with previous research showing that anxious–avoidant pairings are problematic (e.g., Beck et al., 2013), people with greater attachment avoidance likely find the demands for intimacy and emotional intensity of highly anxious individuals to be distressing and overwhelming. At the same time, highly anxious individuals are aware that highly avoidant spouses shy away from their emotional expressions of need. Thus, instead of openly expressing needs and seeking support directly, anxious–avoidant pairings seem to adjust their support-seeking strategies based on their partner’s insecure attachment characteristics (Feeney & Collins, 2001; Simpson et al., 1992). Indirect instrumental support-seeking may reduce relational strain and accommodate the spouse’s insecurity, highlighting the dyadic, adaptive nature of support-seeking in older couples.

We also predicted that older adults who rated their problem as more stressful, as well as those with higher relationship quality, would not seek support indirectly. As expected, older adults with higher relationship quality showed less indirect emotional support-seeking, consistent with our theorizing that it is unnecessary for people to seek support indirectly when they feel comfortable communicating with their spouses and trust their spouse’s ability and willingness to provide responsive support. However, we did not find significant associations between reported stressfulness of the problem and indirect support-seeking in this investigation. This suggests that while increased stress elicits more direct support-seeking, it does not necessarily affect older adults’ simultaneous use of indirect support-seeking strategies.

In sum, we found that both support-seeker’s and support-provider’s attachment insecurity was strongly linked to support-seeker’s indirect support-seeking. Anxious–preoccupied individuals sought emotional support indirectly, possibly driven by their characteristic fear of rejection. Support-seekers also sought instrumental support indirectly from insecurely attached spouses, possibly as a strategic adaptation to their partner’s lack of responsiveness. Conversely, higher relationship quality predicted less indirect emotional support-seeking, suggesting that indirect strategies persist primarily in contexts of interpersonal insecurity.

### 4.3. What Are the Implications for Interventions?

Previous research suggests that direct support-seeking is often the most effective support-seeking strategy, as openly expressing one’s needs allows partners to provide more targeted and helpful support (Barbee & Cunningham, 1995; Don & Hammond, 2017). This is also consistent with the helping literature showing that a critical but often-overlooked predictor of someone providing help is simply being asked to help (Nadler, 2015). The current findings revealing predictors of direct and indirect support-seeking offer insights for designing interventions to promote effective support-seeking in romantic relationships, particularly among older adult couples who may face unique stressors (e.g., health issues, and concerns about losing agency or independence) that require effective mobilization of emotional and instrumental support.

Given that both the support-seeker’s and support-provider’s attachment orientations shape how support is sought, interventions may assist couples in identifying their own and their partner’s attachment tendencies and increasing their state attachment security in support contexts. For example, couples in which both partners are insecurely attached—such as anxious–avoidant pairings—may benefit from interventions that prime attachment security (e.g., exposure to attachment-related words or images) or involve affectionate touch. These simple strategies have been shown to promote state attachment security in close relationships (Gillath et al., 2022; Jakubiak & Feeney, 2016) and may thus encourage more direct support-seeking behaviors and receipt of responsive support in stressful situations.

This study’s findings also highlight that support-seeking is influenced not only by individual dispositions but also by the overall quality of the relationship (e.g., how committed and satisfied people feel within the relationship, and how much they trust their partner). This underscores the value of couple-based interventions designed to improve romantic relationship quality (e.g., couple relationship education programs; Markman et al., 2022), as they help foster a positive relational climate in which direct support-seeking is more likely to occur.

Moreover, the implications extend beyond couple interventions. In caregiving, healthcare, and community settings, where emotional and instrumental support is crucial, interventions could include training caregivers, medical professionals, and program staff to recognize and adapt to the attachment needs of those they care for (Salmoiraghi & Zarotti, 2025). These community-based interventions also could include a variety of relationship-based programs (e.g., peer support programs, and caregiver/staff–patient communication programs) aimed at building strong interpersonal bonds to facilitate support effectiveness. Such strategies could foster open communication, reduce vulnerability, and create a more supportive environment for individuals seeking help.

### 4.4. Contributions and Limitations

This study makes unique contributions to the literature for several reasons. First, it contributes to the relatively sparse literature on support-seeking behaviors in close relationships. Most existing research focuses on support provision but overlooks the important role played by support-seekers in cultivating effective support. This study expands our knowledge of support-seeking in close relationships by informing us that both the support-seeker’s and support-provider’s attachment orientation, as well as the support-seeker’s stress level and relationship quality, are significant predictors of support-seeking behaviors in romantic relationships.

Second, our sample consisted of older adult couples who had been married for over 40 years. While most existing research on support-seeking focuses on undergraduate or young adult couples, little is known about these dynamics in later life. This is a critical gap, as older adults may have a greater need to seek social support due to age-related cognitive and physical declines.

Third, this study employed rigorous methods, including observational data and behavioral coding. Rather than relying on participants’ self-reports of their support-seeking strategies, we observed and coded their natural interactions with their partners in a laboratory setting, while couple members discussed a currently significant life stressor. This approach enabled us to capture more objective and ecologically valid indicators of how support-seeking behaviors unfold in real-time discussions of life stressors.

Several limitations should be addressed in future research. First, this study is correlational in nature; although predictors were measured a week prior to the support-seeking interactions—providing some temporal ordering—causal conclusions cannot be drawn. Experimental studies are needed to test the causal impact of proposed predictors on support-seeking behaviors.

Second, although a strength of this investigation is its focus on an older adult age group that has been neglected in prior work, the results might not generalize to younger couples or couples who have been married or cohabiting for a shorter period of time (who may have less stable interaction patterns). Also, since the study primarily included White Americans and was conducted in the U.S., its findings may not apply to other cultural or geographical contexts. Previous research has shown cultural differences in support-seeking (Lua et al., 2022), such as lower support-seeking behavior in East Asians compared to Westerners. Future research should replicate this work in more diverse age groups, relationship durations, and cultural settings to examine whether predictors of support-seeking strategies differ across populations and relational and cultural contexts.

Third, the inter-rater reliability for indirect instrumental support-seeking was relatively low, reflecting the difficulty of observing and coding subtle or ambiguous indirect support-seeking attempts. Future research could refine the coding system, incorporate facial and syntactic cues, and include additional data sources (e.g., self-reports or partner perceptions) to improve the reliability of these observational assessments.

Finally, this study considers support-seeking only in the context of discussions of one couple member’s personal stressor. There are many other types of social interactions, such as discussions of goals or disagreements that may have different support-seeking processes. We do not currently know if support-seeking will occur in the same way across different types of interactions or if people utilize different support-seeking strategies in different contexts.

## 5. Conclusions

Overall, this work contributes to the sparse literature focusing on support-seeking in close relationships. It provides a unique focus on the aging population, and it employs rigorous methods to systematically identify predictors of distinct support-seeking strategies in close relationships. By shedding light on what motivates individuals to seek support and how they do so, this work advances our understanding of the support-seeking process and lays important groundwork for future research on social support dynamics in later life.

## Figures and Tables

**Table 1:** Descriptive statistics of and correlations among all study variables.

Variable	1	2	3	4	5	6	7	8	9	10
**Support-Seeking**										
1. DISS	—									
2. DESS	0.06	—								
3. IISS	0.14 *	−0.12 ^+^	—							
4. IESS	−0.09	0.18 **	−0.01	—						
**Predictors**										
5. SS Stress	0.17 *	0.01	0.12 ^+^	0.14 ^+^	—					
6. SS Rel. Quality	0.11	−0.07	−0.09	−0.16 *	−0.18 **	—				
7. SS Anxiety	0.06	0.04	−0.05	0.11	0.09	−0.23 ***	—			
8. SS Avoidance	−0.02	−0.09	0.01	−0.05	0.00	−0.24 ***	0.43 ***	—		
9. SP Anxiety	0.10	0.13 ^+^	0.11	−0.03	0.08	−0.10	0.16 *	0.10 ^+^	—	
10. SP Avoidance	0.03	0.21 **	0.08	0.09	0.04	−0.03	0.11 ^+^	0.14 *	0.43 ***	—
Mean	1.34	1.61	1.32	1.51	3.42	−0.01	2.26	3.16	2.35	3.17
SD	0.59	0.63	0.46	0.62	0.96	0.78	0.94	0.95	0.99	0.93

Note: DISS = direct instrumental support-seeking; DESS = direct emotional support-seeking; IISS = indirect instrumental support-seeking; IESS = indirect emotional support-seeking; SS = support-seeker; SP = support-provider. ^+^ *p* < 0.10. * *p* < 0.05. ** *p* < 0.01. *** *p* < 0.001.

**Table 2:** Factors predicting support-seeking.

Predictors	DISS		DESS		IISS		IESS	
	*B (SE)*	*p*	*B (SE)*	*p*	*B (SE)*	*p*	*B (SE)*	*p*
**Step 1**								
SS Stress Level	**0.12 (0.04)**	**0.007**	−0.03 (0.05)	0.548	0.05 (0.03)	0.173	0.06 (0.05)	0.185
SS Attachment Anxiety	0.04 (0.05)	0.433	0.05 (0.05)	0.348	−0.05 (0.04)	0.222	0.08 (0.05)	0.102
SS Attachment Avoidance	0.00 (0.05)	0.991	**−0.11 (0.05)**	**0.030**	0.01 (0.04)	0.871	−0.08 (0.05)	0.084
SS Relationship Quality	**0.13 (0.06)**	**0.022**	−0.09 (0.06)	0.149	−0.05 (0.05)	0.278	**−0.13 (0.06)**	**0.027**
SP Attachment Anxiety	00.07 (0.05)	0.135	0.03 (0.05)	0.489	0.04 (0.04)	0.254	−0.08 (0.05)	0.107
SP Attachment Avoidance	−0.02 (0.05)	0.648	**0.13 (0.05)**	**0.009**	0.02 (0.04)	0.617	0.10 (0.05)	0.052
R^2^	0.07		0.07		0.04		0.07	
**Step 2**								
SS Stress Level × SS Anxiety	0.05 (0.05)	0.344	−0.04 (0.05)	0.479	0.06 (0.04)	0.143	0.02 (0.05)	0.654
SS Stress Level × SS Avoidance	−0.01 (0.04)	0.773	**0.09 (0.04)**	**0.024**	−0.01 (0.03)	0.665	−0.00 (0.04)	0.994
SS Anxiety × SS Avoidance	0.08 (0.04)	0.061	0.02 (0.04)	0.723	−0.01 (0.03)	0.689	**−0.09 (0.04)**	**0.044**
SP Anxiety × SP Avoidance	−0.06 (0.04)	0.089	0.00 (0.04)	0.986	−0.01 (0.03)	0.846	−0.03 (0.04)	0.427
SS Anxiety × SP Anxiety	−0.01 (0.05)	0.809	−0.07 (0.06)	0.240	−0.07 (0.04)	0.093	−0.07 (0.06)	0.224
SS Anxiety × SP Avoidance	−0.03 (0.06)	0.642	−0.06 (0.06)	0.359	**0.09 (0.05)**	**0.041**	0.08 (0.06)	0.212
SS Avoidance × SP Anxiety	−0.01 (0.05)	0.828	0.03 (0.05)	0.598	**0.08 (0.04)**	**0.036**	0.07 (0.05)	0.189
SS Avoidance × SP Avoidance	0.05 (0.06)	0.411	0.05 (0.06)	0.439	−0.06 (0.05)	0.237	−0.09 (0.06)	0.156
R^2^	0.10		0.12		0.08		0.11	

Note: DISS = direct instrumental support-seeking; DESS = direct emotional support-seeking; IISS = indirect instrumental support-seeking; IESS = indirect emotional support-seeking; SS = support-seeker; SP = support-provider.

## Data Availability

The data for this study are available at https://osf.io/mq348/?view_only=e3bc52fc012f4009bd53f8902d057a7e (accessed on 25 August 2025).

## Note

1 Although we did not hypothesize gender differences, we ran the same analyses controlling for gender, and the overall patterns stayed the same. The only significant gender effect was a main effect indicating that women engaged in more direct instrumental support-seeking compared to men.

## Abbreviations

The following abbreviations are used in this manuscript:

DISS	Direct Instrumental Support-Seeking
DESS	Direct Emotional Support-Seeking
IISS	Indirect Instrumental Support-Seeking
IESS	Indirect Emotional Support-Seeking
SS	Support-Seeker
SP	Support-Provider
